# Tilapia viscera hydrolysate extract alleviates oxidative stress and renal damage in deoxycorticosterone acetate-salt-induced hypertension rats

**DOI:** 10.14202/vetworld.2020.2477-2483

**Published:** 2020-11-23

**Authors:** Putut Har Riyadi, Mochammad Fitri Atho’illah, Wendy Alexander Tanod, Irma Sarita Rahmawati

**Affiliations:** 1 Department of Fisheries Post Harvest Technology, Faculty of Fisheries and Marine Science, Diponegoro University, Semarang 1269, Central Java, Indonesia; 2 Department of Biology, Faculty of Mathematics and Natural Science, Brawijaya University, Malang 65145, East Java, Indonesia; 3 Department of Fisheries Product Technology, Institute of Fisheries and Marine (Sekolah Tinggi Perikanan dan Kelautan), Palu 94118, Central Sulawesi, Indonesia; 4 Department of Nutrition, Faculty of Medicine, Brawijaya University, Malang 65145, East Java, Indonesia; 5 Department of Fisheries and Marine Science, Politeknik Negeri Nusa Utara, Tahuna 95821, North Sulawesi, Indonesia

**Keywords:** antioxidant, hydrolysate, peptide, tilapia, viscera

## Abstract

**Background and Aim:**

Hypertension is closely related to oxidative stress conditions, which increases malondialdehyde (MDA) expression and renal damage. Tilapia viscera hydrolysate extract (TVHE) contains compounds and peptides that act as antioxidants. This study aimed to investigate TVHE therapy effect on MDA levels and renal histological conditions in deoxycorticosterone acetate (DOCA)-salt-induced hypertension rats.

**Materials and Methods:**

Tilapia viscera were defatted and hydrolyzed using Alcalase enzyme to obtain TVHE. TVHE antioxidant activity was measured using the 1,1-diphenyl-2-picrylhydrazyl method. Fifteen Wistar male rats were divided into five groups: Normal control (without induced DOCA-salt), DOCA-salt, DOCA-salt+Captopril 5 mg/kg body weight (BW), DOCA-salt+TVHE 150 mg/kg BW, and DOCA-salt+TVHE 300 mg/kg BW. MDA level and renal histology were observed in each group.

**Results:**

TVHE half maximal inhibitory concentration values ranged from 3.87±0.35 μg/mL to 42.03±3.55 μg/mL, which were identified as in the very strong Blois category. TVHE and captopril therapy reduced MDA expression significantly (p<0.05) compared to DOCA-salt only. TVHE and captopril therapy also improved glomerular damage in DOCA-salt-induced hypertension rats.

**Conclusion:**

TVHE has antioxidant ability, decreased MDA level, and decreased glomerular damage in DOCA-salt-induced hypertension rats.

## Introduction

Hypertension is a common risk factor that is closely associated with cardiovascular and renal disease development [1]. Hypertension is a non-communicable disease that represents an increase in blood pressure and prevalence has been increasing since 2010, especially in low- to middle-income countries [2]. Approximately 1 billion people are suspected of hypertension and that number is expected to rise to 1.5 billion by 2025 [3]. Oxidative stress plays a crucial role in hypertension pathogenesis caused by the overproduction of reactive oxygen species (ROS) [4]. Animal models induced by deoxycorticosterone acetate (DOCA)-salt are a hypertensive model that is most characteristic of human cardiovascular remodeling [5]. DOCA-salt increases mineralocorticoid, followed by increased nicotinamide adenine dinucleotide phosphate (NADPH) oxidase activity and superoxide production. This process contributes to excessive oxidative stress formation and a rise in blood pressure [6]. Recent evidence indicates that hypertension is caused by oxidative stress and impaired renal function in DOCA-salt animal models [7].

Several studies have examined antioxidant and antihypertensive peptides from fish products generated by the hydrolysate technique. Products made from raw fish waste, such as chum salmon head [8], tuna fin [9], chum salmon skin [10], yellowtail tuna fin [11], Sardinela head and fins [12], tuna liver [13], skipjack egg [14], turtle egg white [15], bluefin leatherjacket head [16], *bakasang* skipjack [17], and peptides from fisheries waste [18], are known to possess ACE inhibitor activity. These findings indicate that fish residual waste could be a potential antihypertensive source that is nutritionally healthy, safe, cheap, and has less adverse effects due to the natural antioxidant presence [19,20]. Indonesia is the third-largest seafood consumer nation after China and Japan [21] with 91% of aquaculture production [22]. Tilapia is the third largest fish product in Indonesia after carp and salmon [23]. However, fish processing always raised environmental problems, since 50% of the waste material or residue are discarded [24]. Therefore, processing fish residues using the hydrolysis technique can add their nutraceutical and economic value.

Tilapia hydrolysate has many medicinal advantages, including high antioxidant activity in the skin [25]; the viscera suppress intracellular ROS *in vitro* [26]; the skin, bone, frame, head, muscle, and tail have high angiotensin-converting enzyme (ACE) inhibition activity [27,28]; and its viscera also reduce blood pressure, interleukin-6, and tumor necrosis factor-α expression [29]. Our preliminary study showed that tilapia hydrolysate contains adequate necessary amino acids and improved chemical characteristics, which could be met adult human nutritional needs. [30]. However, there is still a lack of information about the tilapia viscera hydrolysate extract (TVHE) effect on oxidative stress and renal damage in hypertensive rats induced by DOCA-salt.

This study investigated the TVHE antioxidant potential on malondialdehyde (MDA) levels and renal histological conditions. This study used DOCA-salt-induced hypertensive rat models to determine the effectiveness of TVHE therapy as a nutraceutical candidate.

## Materials and Methods

### Ethical approval

Animal experiments approved by the Research Ethics Committee, Brawijaya University, Indonesia (Ref. No. 1064-Kep-UB).

### Study period and location

The research was conducted from April to September 2019 at the Experimental Animal Laboratory, Institute of Bioscience, Brawijaya University, Indonesia.

### Sample preparation and extraction

Tilapia viscera were obtained from PT Aquafarm Nusantara, Semarang Industrial Estate, in Indonesia. Viscera were cleaned with water and defatted. The hydrolysis process was carried out using the Alcalase enzyme (Sigma-Aldrich No. 126741) so that TVHE rich in bioactive peptides was obtained. TVHE was optimized using the response surface methodology (RSM) to obtain TVHE with the best hydrolysis degree [31].

### Antioxidant assay

Antioxidant activity was measured using the 1,1-diphenyl-2-picrylhydrazyl (DPPH, Merck) radical scavenging method [32,33]. Twenty-five milligrams of TVHE were added to methanol at an extract concentration of 200 μg/mL. The half maximal inhibitory concentration (IC_50_) was determined using 30, 60, 90, 120, and 150 μg/mL TVHE concentrations. Ascorbic acid (Vitamin C) was used as a comparison control. Two milliliters of each extract solution were added to 2 mL of 50 μM DPPH solution. The solution was homogenized and left for 30 min in a dark room before measuring with a spectrophotometer (Shimadzu UV-1800) at 517 nm absorbance. The DPPH solution absorbance value was also measured as a blank. The DPPH radical scavenging assay was performed in three independent experiments, and the measurement results were expressed as the mean±standard deviation using Microsoft Excel 2016.

The DPPH radical scavenging effect calculated using the equation:


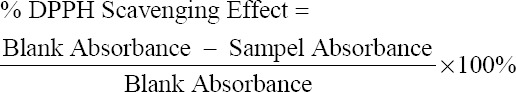


### Animal subject preparations

TVHEs with the best hydrolysis degree were used for therapy in hypertensive Wistar rats induced by DOCA-salt. Fifteen male rats (200-210 g) were adapted for 14 days in a controlled room with 24±2°C, humidity 50-60%, and 12 h dark/light cycle. Rats were given mineral water and standard food (15% crude protein, 12% water content, crude fiber up to 6%, crude fat 3-7%, maximum ash 7%, calcium 0.9-1.1%, and phosphorus 0.6-0.9%). In the pre-treatment phase, DOCA-salt was dissolved in corn oil and orally administered through gavage (except in control rats) 10 times for 5 weeks (2 times a week) to induce hypertension. Two DOCA-salt concentrations were prepared: 20 mg/kg and 10 mg/kg DOCA-salt. At the end of the 5^th^ week, the rats were measured for blood pressure, then divided into five treatment groups: Normal control (without induced DOCA-salt), DOCA-salt, DOCA-salt+Captopril 5 mg/kg of body weight (BW), DOCA-salt+TVHE 150 mg/kg of BW, and DOCA-salt+TVHE 300 mg/kg of BW. Rats were given TVHE and captopril treatments as therapy for 8 days orally through gavage. Systolic blood pressure (SBP) was measured by the tail-cuff method using a blood pressure analyzer. Rats were considered as hypertensive if the SBP was equal to or higher than 150 mmHg [34].

### MDA measurement

Rats were anesthetized with ketamine at the dose of 70 mg/kg BW and dissected to collect the kidney [35]. Kidneys were cut into small pieces and then crushed in a cold mortar on ice. Then, 1 ml of physiological NaCl added. The homogenate was centrifuged at 8000 rpm for 20 min, and the supernatant was taken. The supernatant (100 μL) was diluted by adding 450 μL of distilled water, followed by adding 100 μL trichloroacetic 100%, 250 μL HCl 1 N, and 100 μL Na Thio 1%. The mixture was homogenized using a vortex and centrifuged at 5000 rpm for 15 min. The supernatant was transferred to a new microcentrifuge tube and incubated in a water bath at 100°C for 10 min. The absorbance was measured with a spectrophotometer (Shimadzu UV-1800) at a wavelength of 532.8 nm. After the absorbance data were obtained, the concentrations were calculated using a standard curve.

### Histopathological analysis

Renal tissue histology was carried out according to routine laboratory procedures. After DOCA-salt induction and TVHE therapy, rats were sacrificed using ketamine at the dose of 70 mg/kg BW, and the kidney was taken and fixed with 10% formaldehyde solution for 24 h. The pathological structure was observed by making paraffin renal tissue. After hematoxylin-eosin staining, renal histopathological examination was visualized using an Olympus XC10 camera with a 400×. Renal histopathological tissue images were analyzed using the Olympus Viewer for Imaging Applications program to see cell changes in the renal glomerular and tubular portions.

### Statistical analysis

MDA data are expressed as mean±standard deviation. The Statistical Package for the Social Sciences version 20.0 (IBM Corp., Armonk, NY) was used to analyze the data. All data sets were tested for normal distributions using the Kolmogorov–Smirnov test and were normally distributed. The difference between the groups was analyzed using a one-way analysis of variance followed by the Duncan *post hoc* test. p<0.05 was considered statistically significant.

## Results

### Antioxidant activity of tilapia viscera hydrolyzed extract

**Figure-1:**
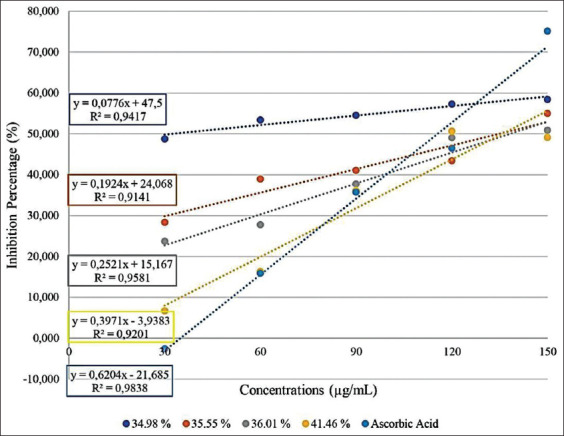
Percentage of 1,1-diphenyl-2-picrylhydrazyl radical inhibition of tilapia viscera hydrolysate extract and ascorbic acid at various concentrations.

The optimization results using the RSM method [31] were obtained for the four TVHEs with the highest degree of hydrolysis, 34.98%, 35.55%, 36.01%, and 41.46%, respectively. The four TVHEs with the highest percentage of hydrolysis were tested for DPPH radical scavenging activity. The IC_50_ results for the four TVHEs are shown in Table-1. The results showed that increased hydrolysis in the TVHEs increased the radical DPPH scavenging percentage in a dose-dependent manner (Figure-1).

**Table-1:** IC_50_ of tilapia viscera TVHE using DPPH radical scavenging method.

Degree of hydrolysis (%)	Mean of IC_50_ (μg/mL)
34.98	42.03±3.55
35.55	13.03±2.28
36.01	8.99±3.36
41.46	3.87±0.35
Ascorbic acid	1.53±0.36

IC_50_=Half maximal inhibitory concentration, TVHE=Tilapia viscera hydrolysate extract, DPPH=1,1-diphenyl-2-picrylhydrazyl

### Therapeutic effect of tilapia viscera TVHE on MDA levels

**Figure-2:**
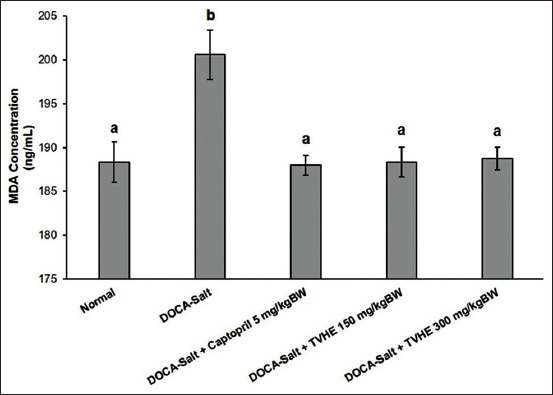
Malondialdehyde concentration in rats treated with deoxycorticosterone acetate (DOCA)-salt, DOCA-salt, and tilapia viscera hydrolysate extract. The values are represented as mean±SD (n=3 for each group). Different letters on the figure considered significantly different for each group at p<0.05 and vice versa based on the Duncan *post hoc* test.

This study demonstrated that DOCA-salt administration in rats significantly increased the concentration of MDA (p<0.05) compared to normal rats (Figure-2). Both administrations of TVHE at 150 and 300 mg/kg of BW significantly reduced the MDA concentration (p<0.05) in DOCA-salt-induced hypertensive rats compared to the DOCA-salt control group. However, these MDA levels were not fully reduced to the normal group MDA levels. Interestingly, there was no substantial difference in MDA concentration in rats treated with captopril and TVHE. These data show that TVHE can induce a therapeutic effect on lower MDA concentrations in DOCA-salt-induced hypertensive rats.

### Therapeutic TVHE effect on renal histopathology

**Figure-3:**
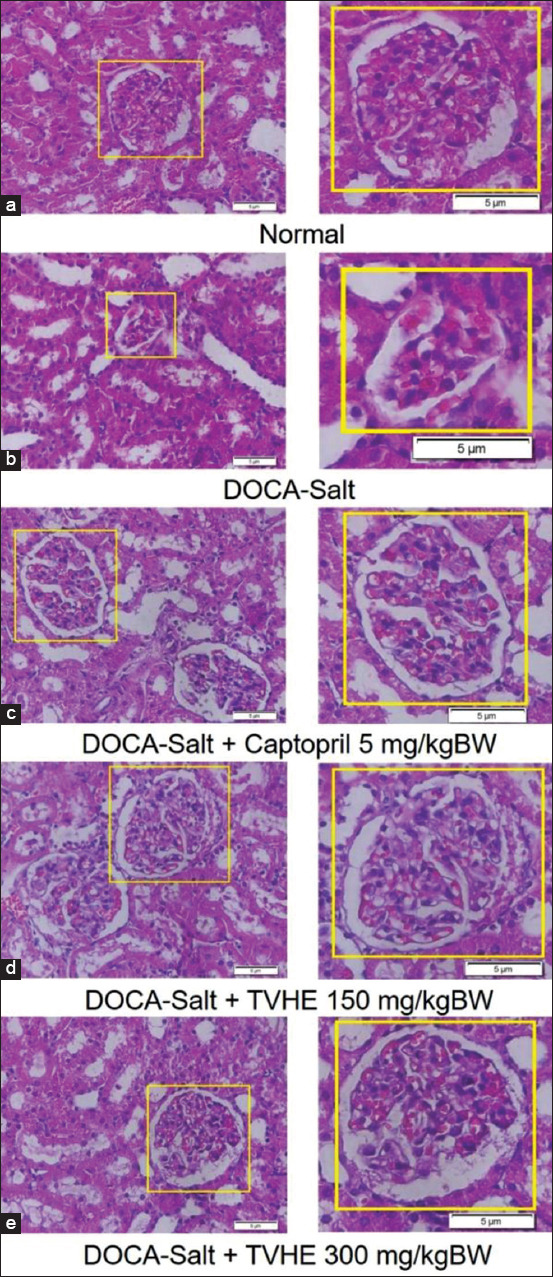
(a-e) Tilapia viscera hydrolysate extract repairs histopathological changes in renal tissues in deoxycorticosterone acetate-salt rats. Renal was stained with hematoxylin and eosin and observed at 400×.

Renal histopathology demonstrated that there were distinct and clear pathological changes, such as necrosis in the glomerular area surrounded by Bowman capsules and renal tubules in DOCA-salt-induced rats compared to normal rat (Figure-3a and b). However, the renal pathology improved in DOCA-salt rats after treated with TVHE (150 and 300 mg/kg of BW) or captopril (5 mg/kg of BW) (Figure-3c-e). The histopathological results showed that TVHE promotes repair in renal pathologies in DOCA-salt-induced hypertension rats.

## Discussion

Using fish by-products processed by the hydrolysis technique have been highlighted in the present year due to their beneficial effects on health. Some of the hydrolysated fish by-products that can act as an antihypertensive are salmon heads, skate skin (*Okamejeri kenojei*), salmon skin, gelatin from thornback ray skin, smooth-hound viscera (*Mustelus mustelus*), tuna fins, meat red tuna, tuna liver, skipjack egg, round Sardinella head and viscera (*Sardinella aurita*), and yellowfin sole bone (*Limanda aspera*) [18]. Our present study demonstrated that TVHE has a strong antioxidant capacity and has beneficial effects on lowering MDA due to increased oxidative stress. This ultimately leads to glomerular repair in hypertensive rats induced by DOCA-salt.

The DPPH antioxidant assay is the most popular tool to evaluate a substance’s ability to inhibit free radicals because it is simple, sensitive, and a fast way to test antioxidants [36,37]. The DPPH reduction measured in the TVHE samples was proportional to the amount of purple solution turning yellow to calculate an IC_50_ value, suggesting that 50% of the DPPH-free radicals are scavenged in solution [33]. Table-1 shows the IC_50_ for the four TVHEs. Blois [38] categorized antioxidant activity: Weak (IC_50_ ranges from 150 to 200 μg/mL); moderate (IC_50_ ranges from 100 to 150 μg/mL); strong (IC_50_ between 50 and 100 μg/mL); and very strong (IC_50_ <50 μg/mL). Based on the Blois category, the TVHE antioxidant ability was very strong. TVHE was almost comparable to ascorbic acid (Vitamin C), an antioxidant substance that is widely used. A previous study reported Vitamin C IC_50_ values ranging from 1.01 to 58.94 μg/mL, depending on the Vitamin C concentration used [32].

Table-1 also shows that the hydrolysis degree was directly proportional to the TVHE IC_50_. This suggests the hydrolysis process increased the peptide number [31,39]. These peptides tend to be short-chain peptides. Simple short-chain peptides can scavenge free radicals with ease [40]. This work suggests the hydrolysis process breaks down TVHE proteins susceptible to oxidation and releasing new peptides in a specific manner. These peptides can be further broken down using enzymes, acids, or bases in the body [41]. This would be advantageous, as shorter and simpler peptide chains have higher antihypertensive potential as bioactive peptides, reducing oxidative stress [29,42].

The hypertension condition causes oxidative stress and produces excess free radicals (ROS) [4]. DOCA-salt-induced rats have endocrine hypertension, which increases oxidative stress [5]. These free radicals increase lipid peroxidation, which breaks down into MDA. MDA is a cellular defect marker caused by free radicals [31,43,44]. Figure-2 shows that DOCA-salt induction caused an increase in MDA expression in rats. DOCA-salt induction increases free radicals, which liberating renal phospholipids. These lipids are further broken down by peroxide to produce MDA [33,45].

Figure-2 also shows that DOCA-salt induction resulted in a significant increase in MDA levels (6.57%). The MDA level increase was related to ROS, was correlated with an increase in blood pressure. NADPH oxidase activation is known to be elevated in the aortic cell membrane at this time, producing ROS in the form of anion superoxide (O_2_^−^) [34]. Increased ROS affects lipid peroxidation and the final product, MDA [35]. Our study suggests that TVHE and captopril therapy reduce MDA levels by 5.90-6.27%. Our result suggests a beneficial effect for captopril and TVHE therapies by reducing the blood pressure and MDA levels in hypertensive rats.

TVHE and captopril may reduce MDA levels by inhibiting ACE, angiotensin-I and angiotensin-II, further reducing aldosterone secretion. Low aldosterone secretion results in decreased blood pressure, NADPH oxidase inactivation, low ROS, and decreased MDA [46]. Our study showed TVHEs ability to reduce antioxidant activity by scavenging free radicals and reducing MDA levels. A previous study treated DOCA-salt rats with 200 mg/kg of bakasang for a week and also reduced MDA levels. Bakasang is a traditional food from Maluku and North Sulawesi made from fermented skipjack tuna [47]. These results showed that fish products have a beneficial effect on reducing MDA levels in DOCA-salt hypertensive rats.

Figure-3 shows the glomerular area surrounded by the Bowman capsules and renal tubules are necrotic in DOCA-salt rats. The necrosis caused the presence of karyolysis in cell nuclei. This is caused by DOCA-salt-induced hypertension, oxidative stress, and subsequent renal damage [35,48]. The TVHE and captopril therapies improved the glomerular tissue structure close to what was observed in normal rats.

TVHE therapy showed antioxidant ability by inhibiting lipid peroxidation, which would further increase enzymatic antioxidants, such as superoxide dismutase in the kidneys. Increased enzymatic antioxidants could decrease the free radical anion superoxide (O_2_^−^) [38,49]. Often, endogenous antioxidants and exogenous antioxidants work together to properly inhibit excessive ROS formation [39,40]. TVHE acts as an exogenous antioxidant that can work by directly donating hydrogen ions to neutralize free radicals’ toxic effects. In addition, TVHE can work indirectly by increasing the endogenous antioxidant expression genes through several mechanisms in the body [50,51].

## Conclusion

TVHE may be an antioxidant that inhibits MDA levels as a consequence of increased oxidative stress in DOCA-salt-induced hypertensive rats. TVHE also demonstrates the ability to restore glomerular tissue damaged due to hypertension in renal tissue histology. However, further investigation is required to assess TVHE bioavailability and efficacy after digestion.

## Authors’ Contributions

PHR has made a significant contribution to conception, design, interpretation of data, drafting, revising the manuscript, and gave final approval of the version to be published. MFA helped in data collection, edited article, and made critical revisions. WAT made a substantial contribution to the acquisition of data, analysis, and drafting of the manuscript. ISR analyzed data, drafted the article, and made a critical revision. All authors read and approved the final manuscript.
